# Benign pneumoperitoneum in newborns: which abdomen to open and which one to observe?

**DOI:** 10.1002/ccr3.569

**Published:** 2016-05-04

**Authors:** Manar Al‐lawama, Hashem M. Al‐Momani, Wael M. AboKwaik, Khaled R. Al‐zaben

**Affiliations:** ^1^ Neonatal‐Perinatal MedicineThe University of JordanAmman11937Jordan; ^2^ Department of SurgeryThe University of JordanAmmanJordan; ^3^ Department of PediatricsThe University of JordanAmmanJordan; ^4^ Department of AnesthesiaThe University of JordanAmmanJordan

**Keywords:** Benign, gastrointestinal perforation, neonate, pneumoperitoneum

## Abstract

Benign pneumoperitoneum in newborns is not a rare condition that should be managed conservatively. Neonatologists and surgical teams should work together to avoid unnecessary and potentially risky procedures.

## Introduction

Neonatal pneumoperitoneum is a well‐known emergency that is usually related to necrotizing enterocolitis in preterm infants 1. Other neonatal gastrointestinal pathologies that cause intestinal perforation (such as congenital anomalies and Hirschsprung's disease) can also cause neonatal pneumoperitoneum 2. The usual treatment is surgical intervention.

Idiopathic pneumoperitoneum in neonates is not a rare condition reported in one series in 6% of cases with pneumoperitoneum 2. It is a benign condition that requires conservative management 3, 4. The criteria by which a surgeon decides on which abdomen to open and which one to observe, is ill‐defined. Thus, increasing the awareness of neonatologists and surgeons about this condition will help decrease complications due to unnecessary procedures.

In this study, we present a case of a newborn who developed pneumoperitoneum. The patient was managed by exploratory laparotomy and was found to have no gastrointestinal perforation, but developed severe complications post surgery. We propose a criterion to differentiate between surgical and benign neonatal pneumoperitoneum.

## Case Report

**Figure 1:**
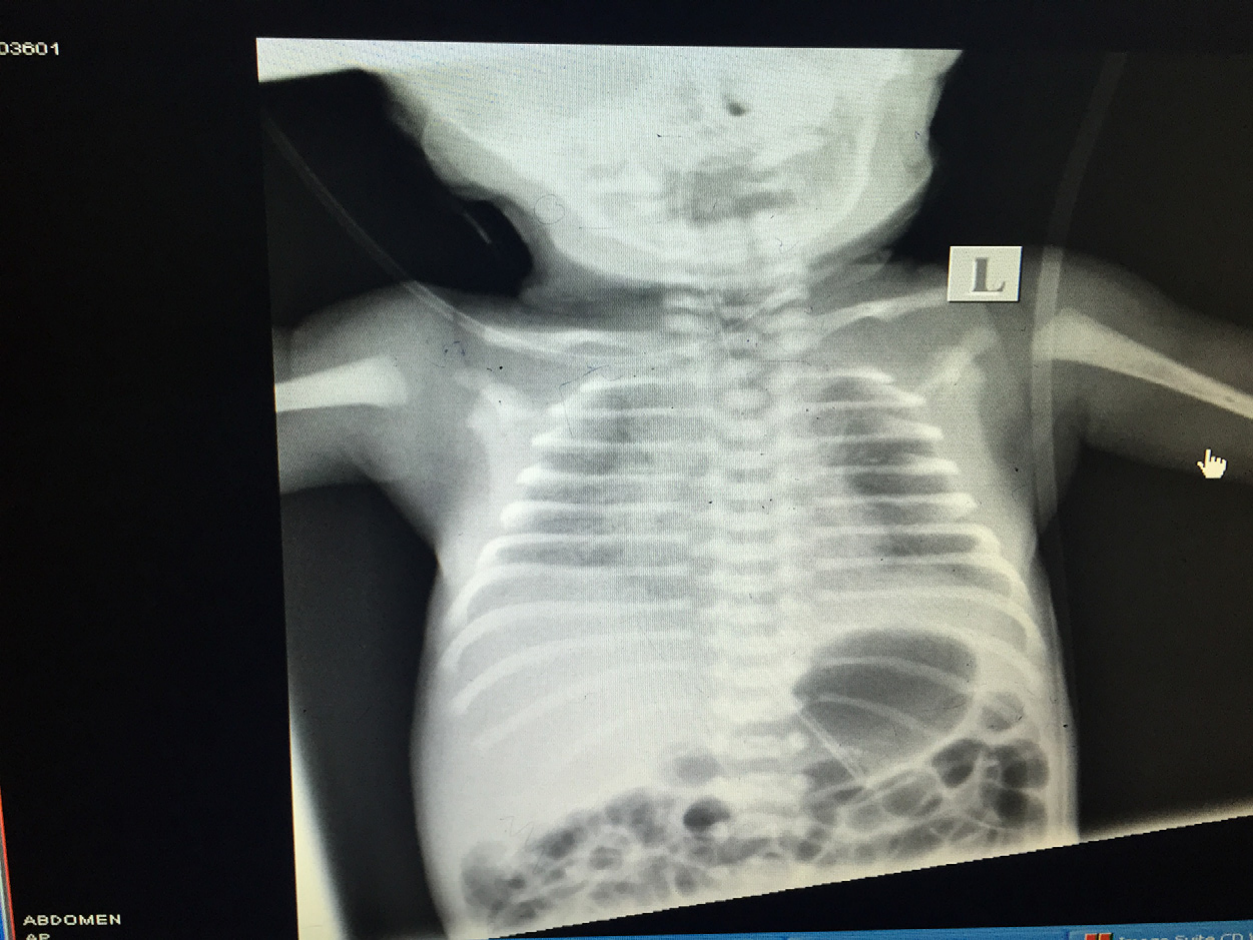
Chest X‐ray showing respiratory distress syndrome changes, No free air is noted in the abdomen.

**Figure 2:**
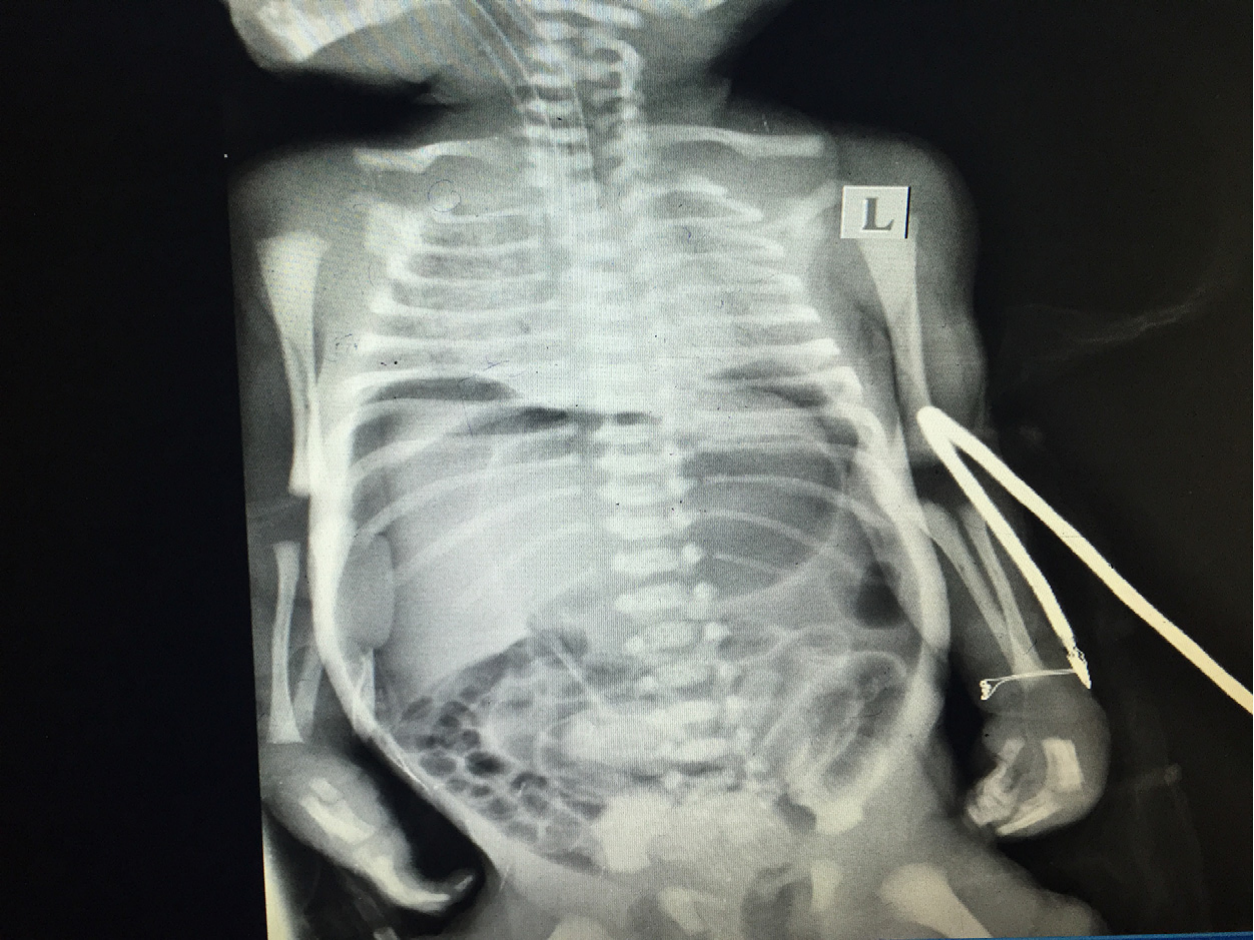
Chest and abdomen X‐ray showing free air in the abdomen.

A 1‐day‐old male newborn (34 weeks of gestation) delivered by cesarean section with a birth weight of 2280 g had APGAR scores of 8 and 9 at 1 and 5 min, respectively. The infant was admitted to the NICU with respiratory distress syndrome and was managed by noninvasive positive pressure ventilation (NIPPV). The chest X‐ray showed severe perihilar infiltrates bilaterally with a fine granular pattern [Fig. 1]. The respiratory status of the infant deteriorated on his second day of life; he required intubation and received surfactant therapy. After surfactant administration, the condition of the infant showed no improvement. A second chest X‐ray was requested, which showed severe lung disease with no evidence of pneumothorax or pneumomediastineum. Free intraabdominal air was noted [Fig. 2]. The abdomen was mildly distended and soft to palpation. Bowel sounds were normal. The temperature, blood pressure, and capillary refill time of the infant were also normal. The laboratory results were all within the normal range except for a low serum calcium level.

Urgent laparotomy was done. There was no gastrointestinal perforation, and the bowels were grossly healthy. A few hours post surgery, the infant developed metabolic acidosis, followed by hypotension and delayed capillary refill. He was managed with inotropes and intravenous hydrocortisone. A head ultrasound was done, which showed bilateral grade 3 intraventricular hemorrhage. The infant had a severe drop in hemoglobin level that necessitated blood transfusion. This was later complicated by posthemorrhagic hydrocephalus that needed VP shunt insertion. The infant also developed a large ductus arteriosus, which responded well to fluid restriction and a 3‐day course of oral paracetamol.

A follow‐up abdominal X‐ray showed no free air in the abdomen. The results of the abdominal examination remained normal, and the infant passed meconium on the second postoperative day. After he was taken off the inotropes, gradual enteral feeding was started, which the infant tolerated very well.

## Discussion

Neonatal pneumoperitoneum with no identified cause is not a rare condition. Increasing the awareness of neonatologists and surgeons about this condition can save newborns from surgical complications and severe neonatal morbidities.

The infant in this study had major postoperative complications, including grade 3 intraventricular hemorrhage and a large ductus arteriosus. Both complications are not common for this gestational age and weight. Although the respiratory condition of the infant might have potentiated his risk for such complications, general anesthesia, hypothermia during surgery, intraoperative fluid boluses, and the transfer process between the NICU and the operating room could have added considerably to the risk of such complications.

Most pneumoperitoneum cases result from gastrointestinal perforation; thus, exploratory laparotomy is most often indicated. However, differentiating benign from surgical pneumoperitoneum might be the key to saving a particular infant from severe morbidities.

We propose, first, that all the nomenclature of the condition be changed from idiopathic or spontaneous pneumoperitoneum to benign pneumoperitoneum in newborns to remind physicians of the management and clinical course pattern of this condition. We further suggest the inclusion of cases in which a thoracic air leak is evident and those that are considered idiopathic because both cases are benign. In addition, we propose a criterion to differentiate those from cases due GI perforation [Table 1].

**Table 1:** Criteria to differentiate benign pneumoperitoneum from pneumoperitoneum due to gastrointestinal perforation in the neonates

	Benign pneumoperitoneum	Gastrointestinal perforation
Birth weight	>1500 g	<1500 g
Gestational age	>32 weeks	<32 weeks
Feed	NPO	Fed
Age at diagnosis	Early < 5 days	Late > 5 days
Antenatal ultrasound	No GI anomalies detected	GI anomalies suspected/No antenatal care
Antenatal NSAID	None	Given
Postnatal steroids	None	Given
Postnatal indomethacin	None	Given
Family history	No history of GI anomalies/Hirschsprung's disease	History of GI anomalies/Hirschsprung's disease
General exam	No dysmorphic features No other congenital anomalies	Dysmorphic features Congenital anomalies present
Peritonitis signs	Absent	Present
Clinical status	Baby status did not deteriorate by the time pneumoperitoneum was detected	Clinical status deteriorated
Pre‐X‐ray diagnosis	Line placement Part of chest X‐ray Mild –moderate abdominal distension	Suspected NEC
X‐ray findings	Absence of Pneumatosis intestinalis No evidence of intestinal obstruction Free air in the abdomen Thoracic air leak	Pneumatosis intestinalis Intestinal obstruction Free air in the abdomen

Neonatologists and surgeons should first focus on the clinical status of the newborns and the results of the abdominal examination. Then, they should try to distinguish between those newborns with risk factors for gastrointestinal perforation and those without. However, newborns without any apparent risk factors can still develop severe gastrointestinal complications. The authors reviewed the literature for cases with idiopathic pneumoperitoneum and for those due to intestinal perforation. Common characteristics for each condition were identified. The proposed criterion is only a guide and a reminder of the process that should be applied to make the right treatment decision for newborn infants.

A gestational age of <32 weeks, a birth weight of <1500 g and enteral feeding are well‐known to be risk factors for necrotizing enterocolitis 5. The presence of any of these should make the medical team suspect necrotizing enterocolitis. Dysmorphic features, congenital anomalies, and antenatal suspicion of gastrointestinal anomalies, all increase the probability that gastrointestinal perforation is the cause of pneumoperitoneum. Antenatal nonsteroidal antiinflammatory drugs have been reported to be a risk factor for intestinal perforation 6. Postnatal steroid and indomethacin therapies have also been associated with gastrointestinal perforation 7. All the reported cases showed no signs of peritonitis 3 and had a relatively stable clinical condition. Most of the patients also had the condition early in life 3.

## Conclusion

Benign pneumoperitoneum in newborns is not a rare condition that should be managed conservatively. Neonatologists and surgical teams should work together to avoid unnecessary and potentially risky procedures. Because there are no diagnostic tests for this condition, the use of the proposed criteria should guide the treating physician when dealing with neonatal pneumoperitoneum.

## Conflict of Interest

None declared.

## Ethical Statement

This is a case report. No patient identification was used. No ethical approval was needed.

## References

[1] Dong, Y. , Y. Q. Xu , and Z. L. Lin . 2015 Clinical analysis of 101 cases of neonatal intestinal perforation. Zhongguo Dang Dai Er Ke Za Zhi 17:113–117. 25760832

[2] Khan, T. R. , J. D. Rawat , I. Ahmed , K. A. Rashid , A. Maletha , M. Wakhlu , et al. 2009 Neonatal pneumoperitoneum: a critical appraisal of its causes and subsequent management from a developing country. Pediatr. Surg. Int. 12:1093–1097. 19844726 10.1007/s00383-009-2488-6

[3] He, T. Z. , C. Xu , Y. Ji , X. Y. Sun , and M. Liu . 2015 Idiopathic neonatal pneumoperitoneum with favorable outcome: a case report and review. World J. Gastroenterol. 20:6417–6421. 26034380 10.3748/wjg.v21.i20.6417 PMC4445122

[4] Zerella, J. T. , and J. Y. McCullough . 1981 Pneumoperitoneum in infants without gastrointestinal perforation. Surgery 89:163–167. 7455901

[5] Fox, T. P. , and C. Godavitarne . 2012 What really causes necrotising enterocolitis? ISRN Gastroenterol. doi:10.5402/2012/628317. 10.5402/2012/628317 PMC3534306 23316377

[6] Kawase, Y. , T. Ishii , H. Arai , and N. Uga . 2006 Gastrointestinal perforation in very low‐birth weight infants. Pediatr. Int. 48:599–603. 17168981 10.1111/j.1442-200X.2006.02282.x

[7] Attridge, J. T. , R. Clark , M. W. Walker , and P. V. Gordon . 2006 New insights into spontaneous intestinal perforation using a national data set: two populations of patients with perforations. J. Perinatol. 26:185–188. 16493433 10.1038/sj.jp.7211439

